# Integrated Navigation Fusion Strategy of INS/UWB for Indoor Carrier Attitude Angle and Position Synchronous Tracking

**DOI:** 10.1155/2014/215303

**Published:** 2014-07-08

**Authors:** Qigao Fan, Yaheng Wu, Jing Hui, Lei Wu, Zhenzhong Yu, Lijuan Zhou

**Affiliations:** ^1^Key Laboratory of Advanced Process Control for Light Industry, Ministry of Education, Jiangnan University, Wuxi 214122, China; ^2^Avlc Radar and Avionics Institute, Wuxi 214122, China

## Abstract

In some GPS failure conditions, positioning for mobile target is difficult. This paper proposed a new method based on INS/UWB for attitude angle and position synchronous tracking of indoor carrier. Firstly, error model of INS/UWB integrated system is built, including error equation of INS and UWB. And combined filtering model of INS/UWB is researched. Simulation results show that the two subsystems are complementary. Secondly, integrated navigation data fusion strategy of INS/UWB based on Kalman filtering theory is proposed. Simulation results show that FAKF method is better than the conventional Kalman filtering. Finally, an indoor experiment platform is established to verify the integrated navigation theory of INS/UWB, which is geared to the needs of coal mine working environment. Static and dynamic positioning results show that the INS/UWB integrated navigation system is stable and real-time, positioning precision meets the requirements of working condition and is better than any independent subsystem.

## 1. Introduction

For many years, positioning technology is developing rapidly. On the ground and in the skies, positioning service can be provided by global positioning systems (GPS) stably and reliably [1, 2]. But in some places, such as coal mine, urban canyons and indoors, due to satellite signal blockage, GPS cannot provide a solution with consistent and long-term stable accuracy. In view of this, indoor positioning becomes a research hotspot.

Whether in the case of indoors or outdoors, inertial navigation system (INS) can output acceleration and attitude angle of carrier synchronously [3, 4]. Yet, it has inherent defects when positioning independence for a long time. Positioning accuracy of INS decreased as the drift error [5]. Generally, GPS is used to provide compensation for INS. But we know that GPS signal is disabled in the case of indoor. Based on the above analysis, we researched ultra wide band (UWB) positioning technique, a kind of effective indoor wireless sensor positioning strategy, as compensation for INS.

UWB is a new advanced and promising positioning technology with centimeter level ranging accuracy and high speed of data transmission characteristics, especially for indoor applications. But in some complicated cases, UWB signal is under the influence of the multipath effect and non-line-of-sight conditions [6, 7]. Ascher et al. [8] presented a tightly coupled UWB/INS system for pedestrian indoor applications and analyzed the influence of integrity monitoring algorithms. Xu et al. [9] researched a new approach using least squares support vector machine and H_*∞*_ filter for integration of INS/WSN and the analysis method is worth using for reference. de Angelis et al. [10] proposed an indoor positioning system based on INS and UWB and described a system solution briefly. Zwirello et al. [11] presented a simulation-based feasibility study on tightly coupled model of UWB and inertial data integration. This approach gives the possibility to profit from UWB measurements even if no direct TDOA solution would be available and step-length-update to smooth the calculated trajectory between consecutive UWB updates. Hol et al. [12] designed a six-DOF tracking system combining UWB measurements with low-cost MEMS inertial measurements. The tracking system not only estimates the position of the sensor unit, but also provides velocity estimates. Evennou and Marx [13] presented an aided dead-reckoning navigation structure and signal processing algorithms for self-localization of an autonomous mobile device by fusing pedestrian dead reckoning and WiFi signal strength measurements, and the system accuracy can be further improved. Tanigawa et al. [14] designed an experimental system of GPS/INS/UWB and studied a multisensor fusion algorithm. Yet, it is only for outdoor use.

From the above, INS/UWB integrated system is a very promising positioning technology. The aim of this paper is to present our work towards closed-loop solution of general indoor positioning based on a combination of INS and UWB distance measurement system. The modeling of INS/UWB integrated system includes system error equation, filtering model, optimal comprehensive strategy, and system experiment. Research results will be used to realize carrier attitude angle and position tracking in indoor environment.

## 2. Error Model of INS/UWB Integrated System

In order to establish the coupling model of INS/UWB, system error needs to be analyzed. Precondition for building the error model of INS/UWB integrated system is to know the error source and transfer rule.

**Figure 1:**
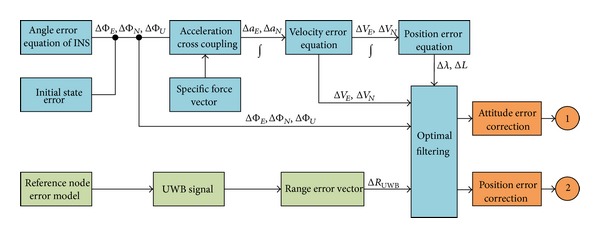
Program plan of multiparameter error propagation and compensation.

As shown in Figure 1, INS error source mainly includes inertial instrument error (gyroscope drift and accelerometer zero bias and measurement noise, etc.), inertial instrument of installation error, system error of initial condition (initial speed of the coal mining machine and position error), system calculation error (integral phase eliminating the high order), and kinds of interference caused by the error. And UWB positioning system error source mainly includes wireless measurement error (multipath diffraction, time delay, and context switching between non-line-of-sight and stadias, etc.), the anchor node coordinate drift, wireless positioning decoding error (nonlinear equations), and node fault. Error transfer rule of INS/UWB is as follows.

- Attitude angle error is produced by angular velocity error through once integral and initial deflection, combined with the state of carrier space error, which forms error angles ΔΦ_*E*_, ΔΦ, and ΔΦ_*U*_. On this basis, coupling the error of acceleration measurement and zero offset, acceleration errors Δ*a*_*E*_ and Δ*a*_*N*_ can be got.
- Further through the integration of the primary and secondary, and combined with the initial position and velocity deviation, we can get velocity errors Δ*V*_*E*_ and Δ*V*_*N*_ and the navigation position errors Δ*λ* and Δ*L*.
- On the one side of the UWB, through the error model, we can obtain the vector error Δ*P*_UWB_ between the reference node and mobile node. On the basis of comprehensive analysis and considering system error transfer relationship, the error equation on all phases is established and the optimal filtering strategy is used; then the correction of inertial attitude, position, and UWB ranging error is completed under the collaborative positioning system.

### 2.1. Error Model of INS

INS error state equation has 13 dimensions, which includes three gyroscope drift errors, three random errors of liner accelerometer, three attitude angle errors, two velocity errors, and two position error states. Selecting ENU coordinate system, we set the gyroscope random error Δ*ε* = [*ε*_*E*_,*ε*_*N*_,*ε*_*U*_]^*T*^ and geographic coordinate of three axial line accelerometer random error ∇_i_ = [∇_E_,∇_N_,∇_U_]^T^; according to the basic error equation of INS [15, 16], we can get the following.

(1) Attitude angle error equation is
(1) ΔΦ˙E=−δVNR1+(VER2tanL+ωesinL)ΔΦN −(VER+ωecos⁡L)ΔΦU+εE,ΔΦ˙N=δVER2−ωesinL−(VER2tanL+ωesinL)ΔΦE −VNR1ΔΦU+εN,ΔΦ˙U=δVER2tanL+(VER2sec2L+ωecos⁡L)ΔL +(VER2+ωecos⁡L)ΔΦE +VNR1ΔΦN+εU.
(2) Velocity error equation is
(2) ΔV˙E=fNΔΦU−fUΔΦN+VNR2ΔVEtanL +(VER2tanL+2ωesinL)ΔΦN −(2ωecos⁡LVE+VEVNR2sec2L)ΔL+∇E,ΔV˙N=fUΔΦN−fEΔΦU −(2ωesinL+VER2ΔVEtanL)ΔΦU −(VE2R2sec2L+2ωecos⁡LVN)ΔL+∇N.
(3) Position error equation is
(3) ΔL˙=ΔVNR1,ΔV˙N=sec LR2ΔVE+VER2ΔL sec LtanL.

Through the error equation, we can analyze the response form of the specific error amount to the particular error factor, and then propagation characteristics of inertial navigation error can be analyzed.

Desirable state vector is

(4) XI=[ΔΦE,ΔΦN,ΔΦU,ΔVE,ΔVN,ΔL,  Δλ,εE,εN,εU,∇E,∇N,∇U]T.

INS system error state equation is

(5) $$\begin{array}{c} {\dot{X}}_{I}\left(t\right)=F_{I}\left(t\right)X_{I}\left(t\right)+W_{I}\left(t\right), \end{array}$$

where  **W**_*I*_(*t*) is the system noise matrix.

**F**_*I*_(*t*) is the system coefficient matrix, which can be represented as follows:

(6) $$\begin{array}{c} F_{I}\left(t\right)={\left(\begin{array}{cc} F_{1}\left(t\right) & F_{2}\left(t\right)\\ 0 & F_{3}\left(t\right) \end{array}\right)}_{13\times 13}, \end{array}$$

where **F**_1_(*t*) is a system matrix which includes 7 related navigation parameters of carrier positioning, and the dimension is 7 × 7. **F**_1_(*t*) = [**F**_1_(*t*)_7×4_, **F**_1_(*t*)_7×3_]:

(7) F1(t)7×4=(VNtanLR2−VUR22ωesinL+VEtanLR20−fU−(2VEtanLR2+2ωesinL)0fU00−1R10ωesinL+VEtanLR21R20−ωesinL−VEtanLR20tanLR20ωecos⁡L+VER2VNR101R100sec LR2000),F1(t)7×3=(fN2ωeVNcos⁡L+VEVNR2sec2L0−fE−VE(VER2sec2L+2ωecos⁡L)0−ωecos⁡L−VER200−VNR1−ωesinL00ωecos⁡L+VER2sec2L00000VER2sec LtanL0),

where **F**_2_(*t*) is the transformation matrix of random error between the gyroscope and accelerometer, and the dimension is 7 × 6:

(8) $$\begin{array}{c} F_{2}\left(t\right)=\left(\begin{array}{cc} O_{2\times 3} & C_{b}^{n}\\ C_{b}^{n} & O_{2\times 3}\\ O_{2\times 3} & O_{2\times 3} \end{array}\right). \end{array}$$

**C**_*b*_^*n*^ is transformation matrix from carrier coordinate system to navigation coordinate system:

(9) $$\begin{array}{c} C_{b}^{n}=\left(\begin{array}{ccc} \cos\varphi \cos\gamma +\sin\varphi \sin\gamma & -\sin\varphi \cos\gamma +\cos\varphi \sin\theta \sin\gamma & -\cos\theta \sin\gamma \\ \sin\varphi \cos\theta & \cos\varphi \cos\theta & \sin\theta \\ \cos\varphi \sin\gamma -\sin\varphi \sin\theta \cos\gamma & -\sin\varphi \sin\gamma -\cos\varphi \sin\theta \cos\gamma & \cos\theta \cos\gamma \end{array}\right). \end{array}$$

And *φ*, *θ*, and *γ* are carrier attitude angles.

Where **F**_3_(*t*) is the system matrix between the gyroscope and the accelerometer random error and the dimension is 6 × 6, *T*_*g*_ and *T*_*a*_ are time constant of gyroscope and accelerometer error, respectively:

(10) $$\begin{array}{c} F_{3}\left(t\right)=\mathrm{diag}\left[\frac{-1}{T_{gx}},\frac{-1}{T_{gy}},\frac{-1}{T_{gz}},\frac{-1}{T_{ax}},\frac{-1}{T_{ay}},\frac{-1}{T_{az}}\right]. \end{array}$$

We carried on the simulation about three main factors affecting inertial navigation positioning, which are the acceleration drift, the gyroscope drift, and the environmental noise, respectively. We added the sensor error to the coordinate positioning system of carrier, as shown in Table 1.

**Figure 2:**
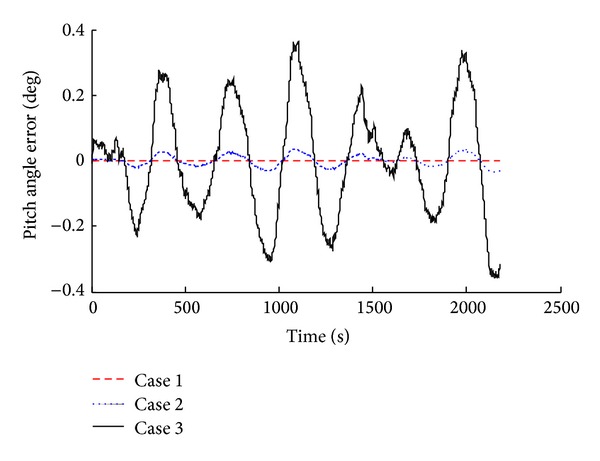
Pitch angle errors with different sensor error.

Simulation results were compared and analyzed, as shown in Figure 2 to Figure 7.

**Figure 3:**
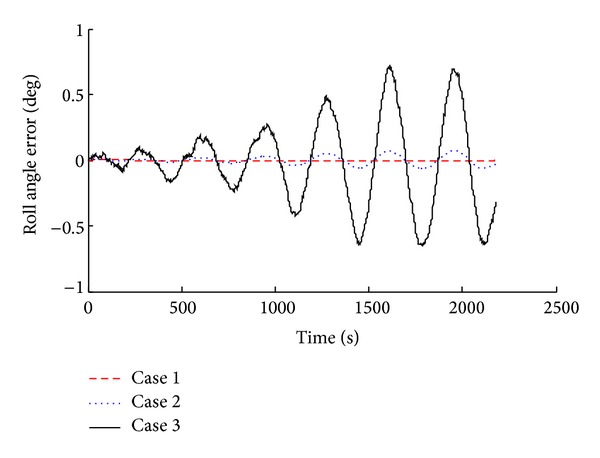
Roll angle error with different sensor error.

**Figure 4:**
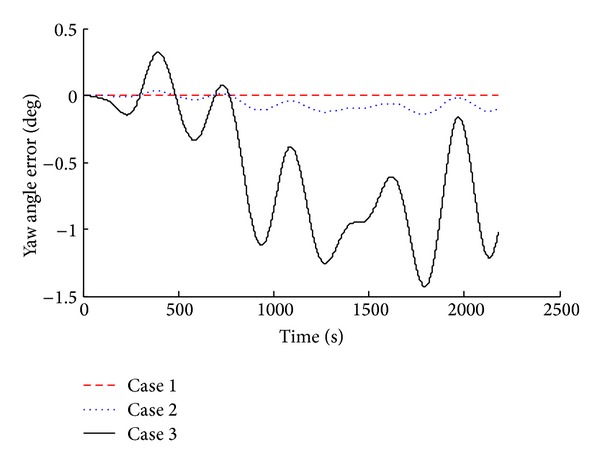
Yaw angle error with different sensor error.

**Figure 5:**
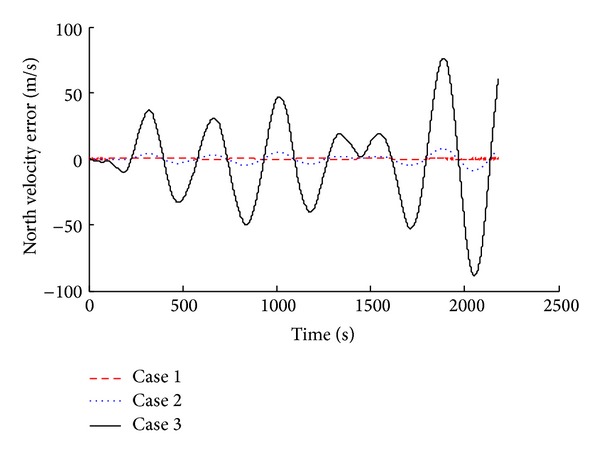
North velocity error with different sensor error.

**Figure 6:**
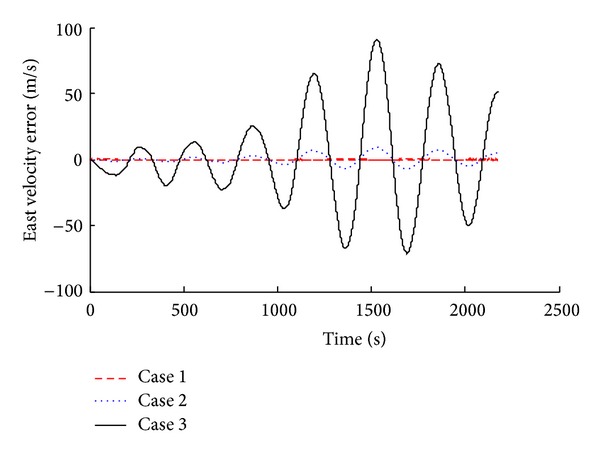
East velocity error with different sensor error.

**Figure 7:**
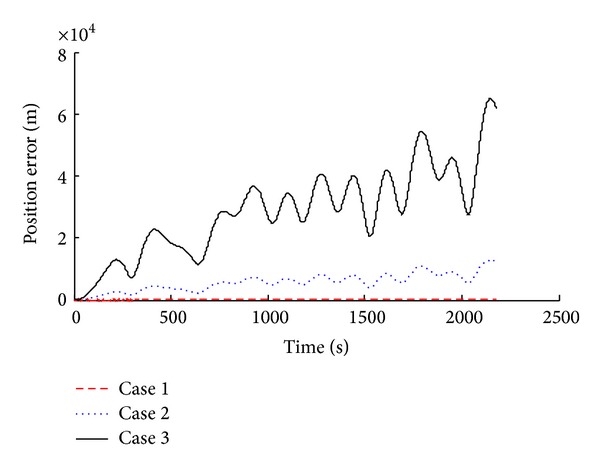
Position error with different sensor error.

The simulation results show that when we consider the drift of inertial device in SINS, corresponding system error increases. According to the attitude angle error results (Figures 2, 3, and 4), we know that, with the inertial device drift and random noise increasing, the attitude angle error of carrier amplitude increases, but it does not show the tendency of divergence. Overall angle error is controlled within ±2° and the maximum of yaw angle error is −1.491°. Because of acceleration and gyroscope drift, the velocity error amplitude increases rapidly. As shown in Figures 5 and 6, the maximum value of northern velocity is −98.3 m/s, and, according to the speed integral of the carrier, we know that there is divergence feature for positioning error obviously, as shown in Figure 7, which embodies the imperfection of using the pure inertial navigation system for carrier location; namely, due to the inertial device drift and random error, location information will cause serious losing of the reliability within a short time and will be not suitable for the carrier positioning.

### 2.2. Error Model of UWB

**Figure 8:**
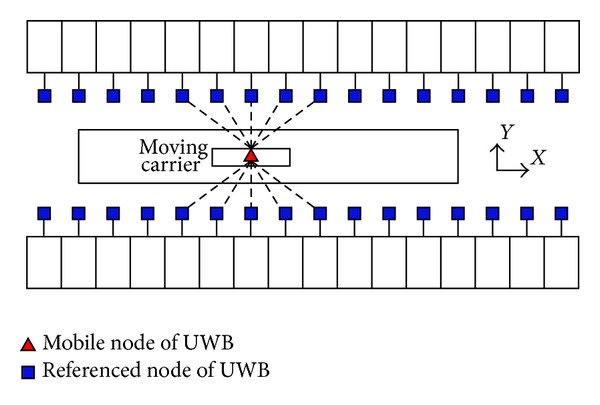
Node arrangement based on UWB in the enclosed environment.

In many of the enclosed environment applications, UWB positioning is two-dimensional plane, and one example is the UWB positioning model shown in Figure 8.

**Figure 9:**
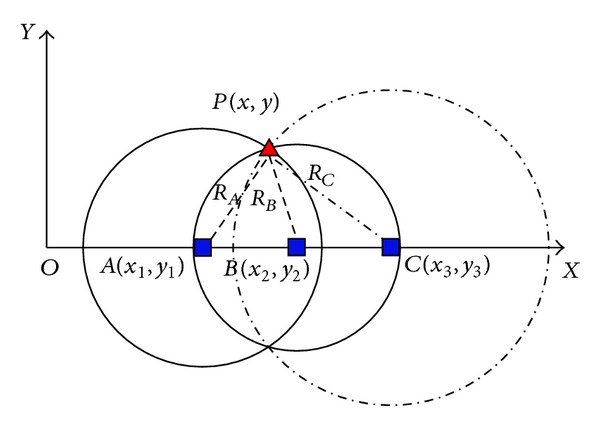
Positioning model of the carrier based on UWB.

Precondition of the error analysis for UWB positioning system is establishing the relationship between the arrival time TOA error and circle location line error. As shown in Figure 9, given the position line equation of UWB:

(11) $$\begin{array}{c} R_{A}=\sqrt{{\left(x-x_{1}\right)}^{2}+{\left(y-y_{1}\right)}^{2}},\\ R_{B}=\sqrt{{\left(x-x_{2}\right)}^{2}+{\left(y-y_{2}\right)}^{2}}, \end{array}$$

(12) $$\begin{array}{c} R_{A}=cT_{oA},\\ R_{B}=cT_{oB}, \end{array}$$

where *T*_*oA*_ and *T*_*oB*_ are time differences of arrival of *A*(*x*_1_, *y*_1_) and *B*(*x*_2_, *y*_2_), respectively, mobile node of carrier *P*(*x*, *y*) can be got by (11).

Definition of position line error is the vertical distance between the line of real position and the line of measuring position. Based on this, relationship between positioning error parameter Δ*u* and position line error Δ*R* can be described as follows:

(13) $$\begin{array}{c} \Delta R=\frac{\Delta u}{\sqrt{{\left(\partial u/\partial x\right)}^{2}+{\left(\partial u/\partial y\right)}^{2}}}=c\Delta t, \end{array}$$

where Δ*t* is TOA arrival time error. Combined with (11), the location of the line equation is obtained, so, through the differential calculation with *x*,  *y*, *R*_*A*_, *R*_*B*_, and *R*_*C*_, we get

(14) $$\begin{array}{c} R_{A}\Delta R_{A}=\left(x-x_{1}\right)\Delta x+\left(y-y_{1}\right)\Delta y,\\ R_{B}\Delta R_{B}=\left(x-x_{2}\right)\Delta x+\left(y-y_{2}\right)\Delta y,\\ \Delta x=\frac{\left(y-y_{1}\right)R_{A}\Delta R_{A}-\left(y-y_{1}\right)R_{B}\Delta R_{B}}{\left(x-x_{1}\right)\left(y-y_{2}\right)-\left(x-x_{2}\right)\left(y-y_{1}\right)},\\ \Delta y=\frac{\left(x-x_{1}\right)R_{A}\Delta R_{A}-\left(x-x_{1}\right)R_{B}\Delta R_{B}}{\left(x-x_{2}\right)\left(y-y_{1}\right)-\left(x-x_{1}\right)\left(y-y_{2}\right)}. \end{array}$$

In the process of positioning, airborne movement node can receive signal, which is more than three reference nodes. If Δ*t* is zero, *N* equations of position line will meet a point, at the same time, according to observation equations determined in the abovementioned model, and then we can obtain no fuzzy solution of shearer location coordinates. But error is inevitable in practice system; the line of circle position formed by these reference points cannot meet a point; the position to solve the problem is evolved into overdetermined equations to solve the problem. Caffery positioning method [17, 18] is used in the paper. This paper will transform nonlinear equations of round position line into linear equations and then using the least-square method estimate airborne movement node location.

When *N* airborne mobile node signals are received and *N* nonlinear equations on carrier position are given, by subtracting the *N*− 1 equation from the *N* equation,

(15) 12(R12−R22+x22−x12+y22−y12)  =(x2−x1)x+(y2−y1)y,12(R22−R32+x32−x22+y32−y22)  =(x3−x2)x+(y3−y2)y,⋮12(RN−12−RN2+xN2−xN−12+yN2−yN−12)  =(xN−xN−1)x+(yN−yN−1)y.

After the transformation, we can make the *N* circle position line equations into *N* − 1 and then make the *N* nonlinear equations into *N* − 1. By selecting suitable reference node, further least-square solutions are got:

(16) $$\begin{array}{c} x=\;{\left(A^{T}A\right)}^{-1}A^{T}b, \end{array}$$

where

(17) $$\begin{array}{c} A=\left(\begin{array}{cc} x_{2}-x_{1} & y_{2}-y_{1}\\ x_{3}-x_{2} & y_{3}-y_{2}\\ \vdots & \vdots \\ x_{N}-x_{N-1} & y_{N}-y_{N-1} \end{array}\right),\\ b=\frac{1}{2}\left(\begin{array}{c} R_{1}^{2}-R_{2}^{2}+x_{2}^{2}-x_{1}^{2}+y_{2}^{2}-y_{1}^{2}\\ R_{2}^{2}-R_{3}^{2}+x_{3}^{2}-x_{2}^{2}+y_{3}^{2}-y_{1}^{2}\\ \vdots \\ R_{N-1}^{2}-R_{N}^{2}+x_{N}^{2}-x_{N-1}^{2}+y_{N}^{2}-y_{N-1}^{2} \end{array}\right),\\ x=\left(\begin{array}{c} x\\ y \end{array}\right). \end{array}$$

When parameter measurement error of UWB occurred, according to the abovementioned equations, goal error can be described as

(18) $$\begin{array}{c} \Delta x=\left(\begin{array}{c} \Delta x\\ \Delta y \end{array}\right)={\left(A^{T}A\right)}^{-1}A^{T}BR\circ \Delta R, \end{array}$$

where ∘ is Schur product, **B** is coefficient matrix of the position line,  **R** is the distance between mobile node and reference node, and Δ**R** is the distance error. The parameter of each reference node is independent. Parameter equation is *δ*_*R*_^2^. Then we can determine the positioning covariance as follows:

(19) $$\begin{array}{c} P_{d}=\delta_{R}^{2}\left[{\left(A^{T}A\right)}^{-1}A^{T}B\right]D{\left[{\left(A^{T}A\right)}^{-1}A^{T}B\right]}^{T}, \end{array}$$

where **D** = diag⁡(*R*_1_^2^, *R*_2_^2^,…, *R*_*N*_^2^) and **D** is given by the coordinate estimation.

### 2.3. Combined Filtering Model of INS/UWB

**Figure 10:**
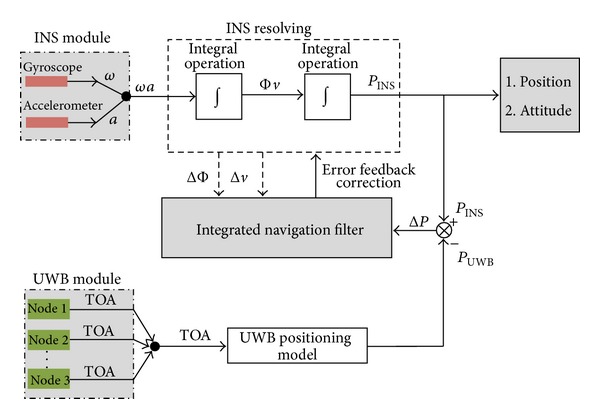
Coupled model diagram of INS and UWB.

INS and UWB are two separate systems and the coupling model is built in order to make the complementary advantages, as shown in Figure 10. According to the coupling model, INS/UWB integrated navigation is realized by using feedback correction method in this paper. On the one hand, attitude and velocity error of INS are input to the Kalman filter. On the other hand, position error can be got by difference of output parameters between INS and UWB. Based on the estimates value of attitude error, velocity error, and position error, output correction is made for the navigation parameters.

In the INS/UWB integrated navigation system, the real-time location given by INS is geographic longitude and latitude, while UWB is relative positioning information. Defining *L*_INS_ as the latitude output by INS and *λ*_INS_ as the longitude, *L* and *λ* are the real value, respectively. Then, location information can be represented as

(20) $$\begin{array}{c} L_{\text{INS}}=L+\Delta L,\\ \lambda_{\text{INS}}=\lambda +\Delta \lambda . \end{array}$$

At the same time, Δ*R*_*x*_ and Δ*R*_*y*_ are eastern and northern measurement distance error of UWB, respectively, after coordinate transformation, longitude, and latitude output of UWB are *L*_UWB_ and *λ*_UWB_, respectively:

(21) $$\begin{array}{c} L_{\text{UWB}}=L-\frac{\Delta R_{y}}{R_{1}},\\ \lambda_{\text{UWB}}=\lambda -\frac{\Delta R_{x}}{R_{2}\cos L}, \end{array}$$

where *R*_1_ and *R*_2_ are the earth ellipsoid local meridian and the local prime vertical curvature radius, respectively. Taking location difference as observation measurement, then location observation equation can be represented:

(22) $$\begin{array}{c} Z_{k}\left(t\right)=\left[\begin{array}{c} \left(L_{\text{INS}}-L_{\text{UWB}}\right)R_{1}\\ \left(\lambda_{\text{INS}}-\lambda_{\text{UWB}}\right)R_{2}\cos L \end{array}\right]=\left[\begin{array}{c} R_{1}\Delta L+\Delta R_{y}\\ R_{2}\Delta \lambda \cos L+\Delta R_{x} \end{array}\right]. \end{array}$$

We can get

(23) $$\begin{array}{c} Z_{k}\left(t\right)=J_{k}\left(t\right)X_{k}\left(t\right)+V_{k}\left(t\right), \end{array}$$

where

(24) $$\begin{array}{c} J_{k}\left(t\right)=\left[O_{2\times 5},\mathrm{diag}\left[R_{1},R_{2}\cos L\right],O_{2\times 3}\right],\\ V_{k}\left(t\right)=\left[\Delta R_{y},\Delta R_{x}\right]. \end{array}$$

As shown in (20), we can get the measurement equation of integrated system. Then, in this paper, optimal filtering strategy of INS/UWB will be researched.

## 3. Optimal Comprehensive and Filtering Strategy of INS/UWB

In view of the closed environment, INS/UWB integrated navigation strategy is adopted to synchronous detection of the carrier, which is a typical multisensor information fusion problem. The core is integrated navigation data filtering fusion. In this field, the most widely used and most successful information fusion technology is Kalman filter [19]. In this paper, integrated navigation data fusion strategy of INS/UWB based on Kalman filtering theory was researched.

### 3.1. Information Fusion Method

Traditional Kalman filter (KF) is an optimal estimation algorithm which is linear, unbiased, and taking minimum error variance for estimation criterion [20]. When the system equation of INS/UWB is known, at the same time, on the condition of the system noise and measurement noise statistical properties known, using linear Kalman filtering technology, the optimal estimate can be realized. But dynamic positioning system in closed environment has time-varying characteristics and the state statistical feature of measurement noise is unknown. If traditional linear Kalman filtering strategy is used directly, filtering precision will be down rapidly, even divergence. In order to solve this problem, fuzzy adaptive Kalman filtering (FAKF) is proposed in this paper, and its purpose is to ignore the accurate measurement noise prior data in the process of filtering. On the basis of the classical Kalman recursive equations, add the measurement noise regulation equation:

(25) $$\begin{array}{c} M_{k}=c_{k}^{d}M_{k-1}, \end{array}$$

where  **M**_*k*_ is the *k* step measurement noise estimation, *c*_*k*_^*d*^ is the adjustment coefficient of measurement noise, and *d* has a great influence for *c*_*k*_^*d*^. When *d* = 0, at this point the measurement noise is not needed to be adjusted; when *d* < 1, the adjustment range is small and the cycle is longer, but the process is stable; when *d* > 1, the adjustment range is larger and cycle is shorter, but it is easier to generate oscillation. *c*_*k*_ can be obtained by fuzzy inference system (FIS) [21] and input reference of FIS is got by the difference between residual observed value and estimate value with INS/UWB measurement model. Defining *r* as the measurement residual, *T*_*r*_ as measurement variance, and *V*_*r*_ as estimating equations, combined with (23), we can get

(26) $$\begin{array}{c} r_{k}=Z_{k}\left(t\right)-{\hat{Z}}_{k}\left(t\right)=Z_{k}\left(t\right)-H_{k}{\hat{X}}_{k\;|\;k-1}, \end{array}$$

(27) $$\begin{array}{c} T_{r}=\frac{1}{N}\overset{k}{\underset{i=i_{0}}{\sum }}r_{i}r_{i}^{T}, \end{array}$$

(28) $$\begin{array}{c} V_{r}=M_{k-1}+H_{k}\left(\Phi_{k,k-1}P_{k-1}\Phi_{k,k-1}^{T}+Q\right)H_{k}^{T}. \end{array}$$

As shown in (26), where  **Z**_*k*_(*t*) is practical measurement value of INS/UWB system, ${\hat{Z}}_{k}(t)$ is measurement estimate value. In (27), *i*_0_ = *k* − *N* + 1. Defining *PR* as the difference value between residual measured variance and estimated variance, there is

(29) $$\begin{array}{c} PR\left(k\right)=\;T_{r}\left(k\right)-V_{r}\left(k\right). \end{array}$$

**Figure 11:**
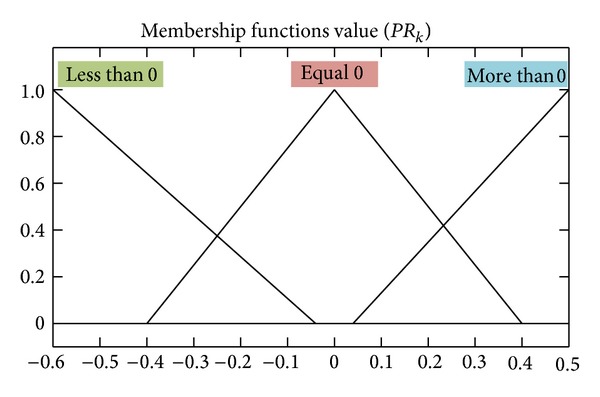
The membership function of the FIS input* PR*_*k*_.

**Figure 12:**
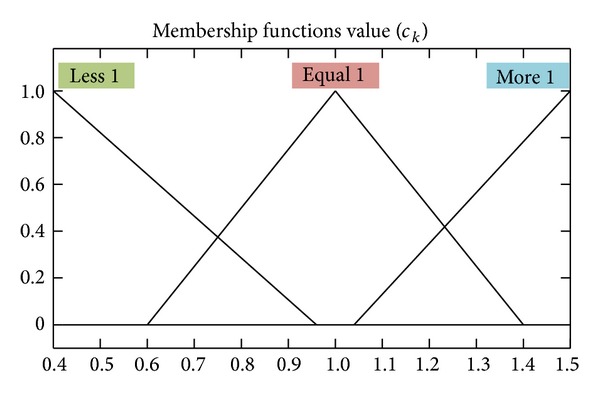
The membership function of the FIS input* c*_*k*_.

On the condition of constructing accurately system model, *PR*(*k*) should be zero; namely, the residual actual variance and theoretical variance are equal. If system noise increases, *T*_*r*_ will increase, *PR*(*k*) > 0, at this time, and **M**_*k*−1_ will increase, which makes *PR*(*k*) close to zero. If system noise reduces, *T*_*r*_ will decrease, and *PR*(*k*) < 0, at this time, and **M**_*k*−1_ will reduce; then *PR*(*k*) will be close to zero. As shown in (25), if *c*_*k*_ > 1, **M**_*k*−1_ will increase; if *c*_*k*_ < 1, **M**_*k*−1_ will reduce; if *c*_*k*_ = 1, **M**_*k*−1_ will not be changed. Further, by setting the fuzzy rules, *PR*(*k*) and *c*_*k*_ will be given, as shown in Figures 11 and 12.

### 3.2. Simulation Research of INS/UWB Based on Fuzzy Adaptive Kalman Filtering

Based on the INS/UWB coupling mechanism, the state of integrated navigation system is

(30) $$\begin{array}{c} X={\left[\Delta \Phi_{E},\Delta \Phi_{N},\Delta \Phi_{U},\Delta V_{E},\Delta V_{N},\Delta X,\Delta Y\right]}^{T}, \end{array}$$

where ΔΦ_*E*_, ΔΦ_*N*_, and ΔΦ_*U*_ are the imbalance angle of east, north, and up direction, respectively, where Δ*V*_*E*_ and Δ*V*_*N*_ are the velocity error of east and north direction, respectively, and where Δ*X* and Δ*Y* are latitude error and longitude error, respectively, by coordinate transformation; then the measure vector of integrated system is

(31) $$\begin{array}{c} Z=\left[\Delta V_{E},\Delta V_{N},\Delta X,\Delta Y\right]. \end{array}$$

**Figure 13:**
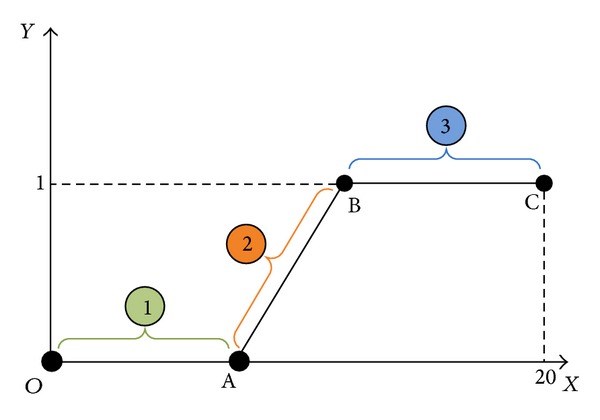
Simulation trajectory schematic diagram.

**Figure 14:**
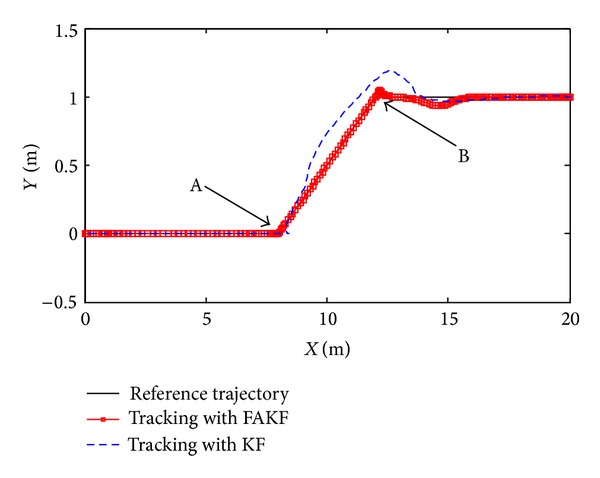
Trajectory tracking effect comparison between FAKF and KF.

**Figure 15:**
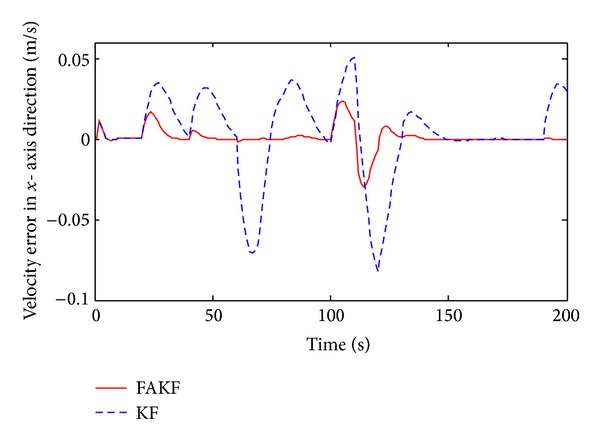
Velocity error in *x*-axis direction.

**Figure 16:**
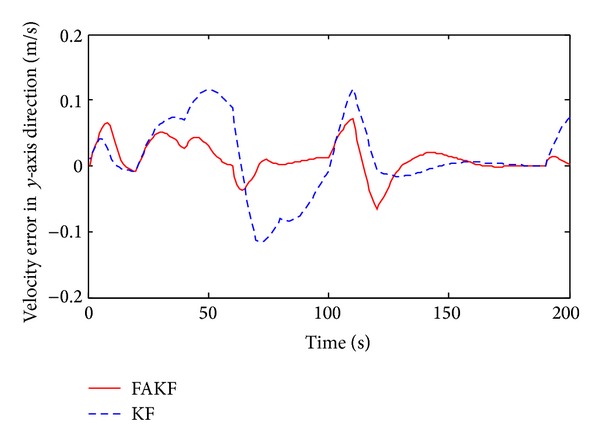
Velocity error in *y*-axis direction.

**Figure 17:**
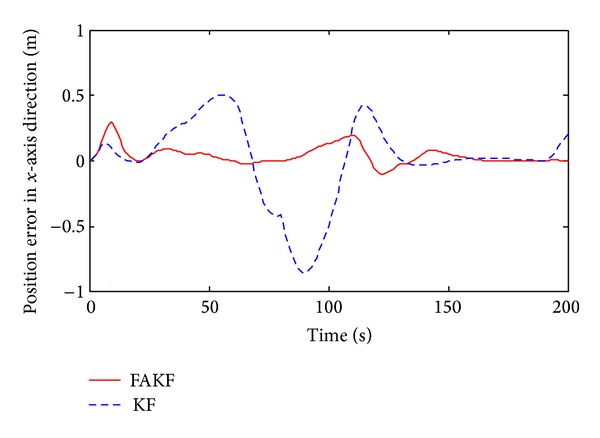
Position error in *x*-axis direction.

**Figure 18:**
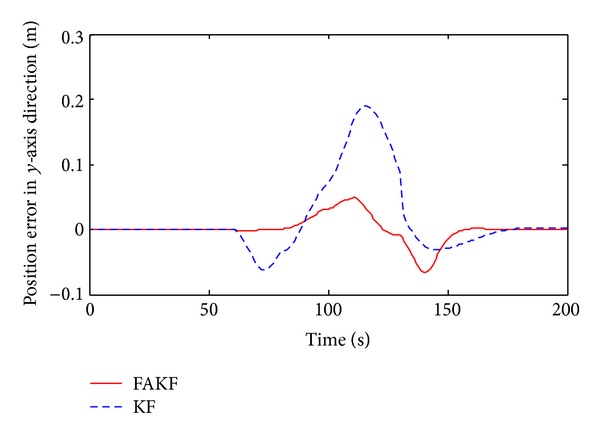
Position error in *y*-axis direction.

As shown in Figure 13, we set up simulation test scenarios. Simulation trajectory contains straight line and broken line. The simulation time is 200 s and the initial position is *P*_*O*_ = (0,0). In the first stage, the initial acceleration on the *X* direction is 0.01 m/s^2^ and the *y*-axis direction is zero. After 90 seconds into the second stage, at point A, acceleration on the *X* direction keeps at 0.01 m/s^2^ and on the *Y* direction becomes 0.0025 m/s^2^; lasting time is 60 s to 110 s; this process is at the end of the point B. Then entering the final stage, the moving target makes a linear motion along the *x*-axis and the lasting time is 90 s. Simulation results are shown in Figures 14, 15, 16, 17, and 18. Initial system noise is *Q*_0_ = diag[2 × 10^−3^, 2 × 10^−3^, 2 × 10^−3^, 1 × 10^−3^, 1 × 10^−3^, 1.5 × 10^−3^, 1.5 × 10^−3^] and the initial measurement noise is *R*_0_ = diag[1 × 10^−4^, 1 × 10^−4^, 2 × 10^−4^, 2 × 10^−4^].

By the results, we can know that in the period of 70 s~140 s measurement error is larger than other periods and the tracking error of KF increases obviously. Yet, FAKF is able to adjust the measurement noise and reduce the tracking error. We make a comparison result, as shown in Table 2. Results show that the adaptive fuzzy Kalman filter method is better than the conventional Kalman filtering, because of smaller tracking error, which is suitable for INS/UWB integrated navigation data fusion.

## 4. Experimental Researches

**Figure 19:**
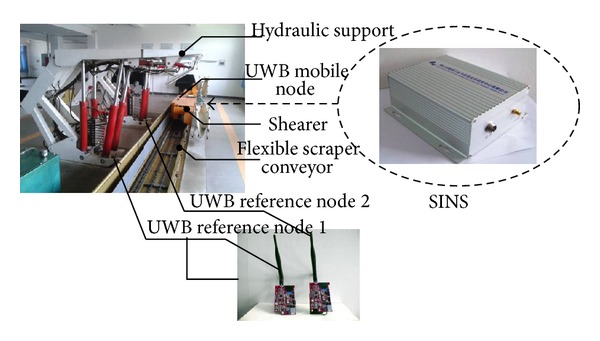
The positioning test platform of combined navigation with INS and UWB.

**Figure 20:**
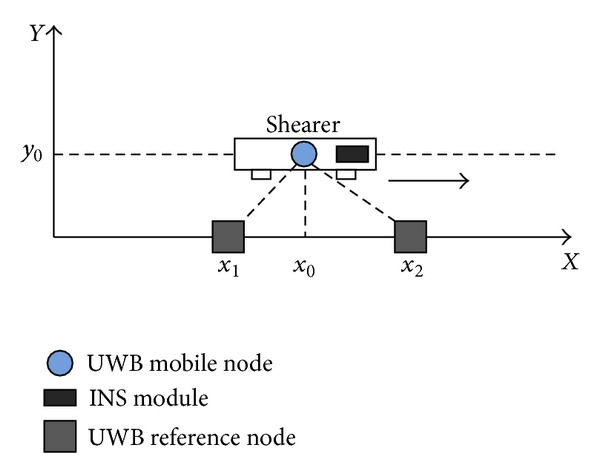
Coordinate orientation diagram.

An indoor experiment platform is established in order to verify theoretical model. The experiment platform is geared to the need of coal mine environment, which is built by the ratio of 1 : 3 scales relative to the actual working condition. Composition mainly includes shearer, hydraulic support, scraper conveyor, INS module, and UWB module, as shown in Figure 19. Then, as shown in Figure 20, two reference node coordinates are set: (*x*_1_, *y*_1_) = (2, 0) and (*x*_2_, *y*_2_) = (4, 0); *Y* axis is *y*_0_ = 1. Based on the integrated model of INS/UWB, the static and dynamic positioning tests are researched.

### 4.1. Static Positioning Test

**Figure 21:**
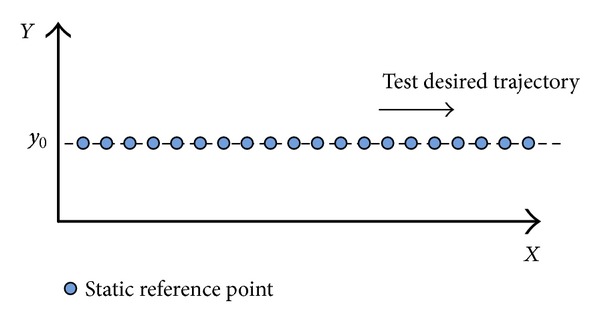
Reference point layout for static positioning test.

As shown in Figure 21, static reference point is set in interval of 1 m, a total of 20 basis points. Results are shown in Table 3; *x*_*R*_ and *y*_*R*_ are *X* and *Y* position of static reference point, respectively, *x*_UWB_ and *y*_UWB_ are output by UWB; *x*_INS/UWB_ and *y*_INS/UWB_ are output by INS/UWB.

**Figure 22:**
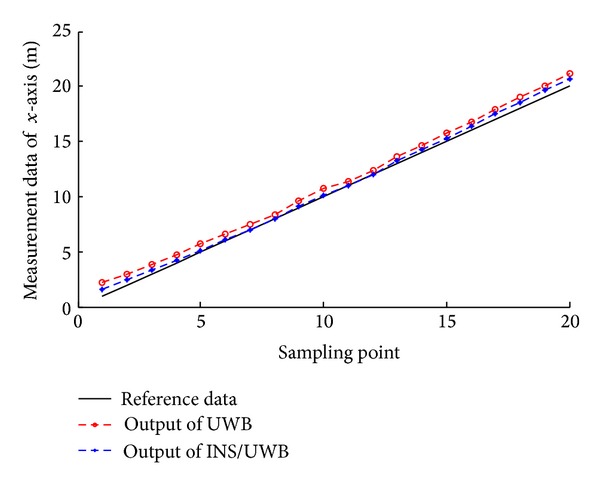
Static tracking effect of *X* coordinate.

**Figure 23:**
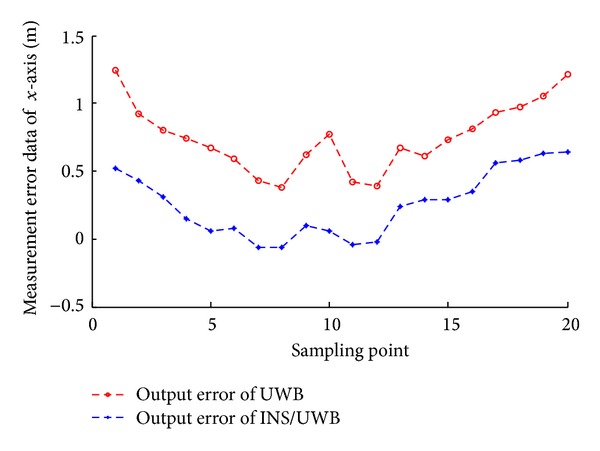
Static tracking error of *X* coordinate.

**Figure 24:**
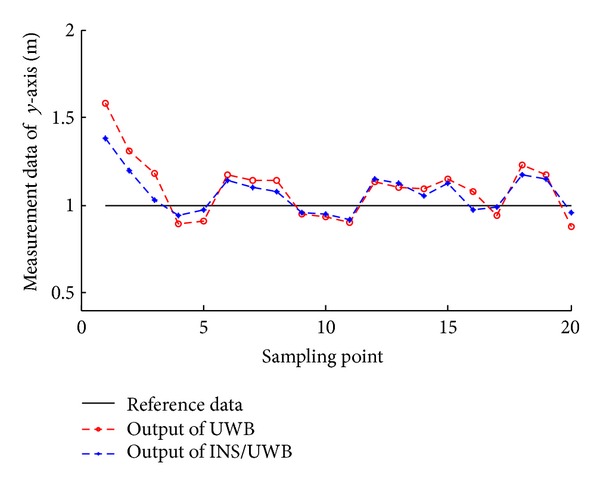
Static tracking effect of *Y* coordinate.

**Figure 25:**
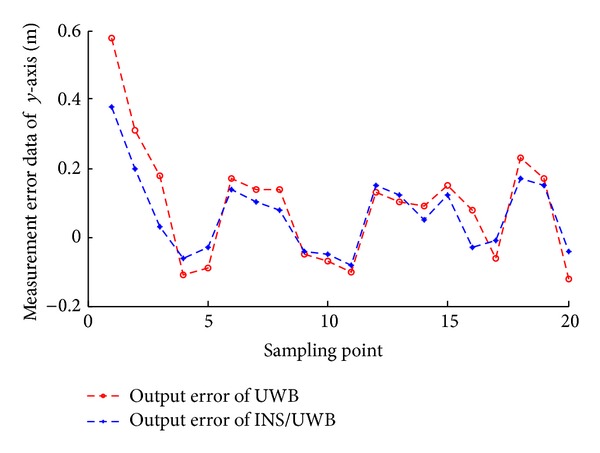
Static tracking error of *Y* coordinate.

According to the measuring data, we can get the track renderings of *X* and *Y* direction, respectively, as shown in Figures 22, 23, 24, and 25.

### 4.2. Dynamic Positioning Test

**Figure 26:**
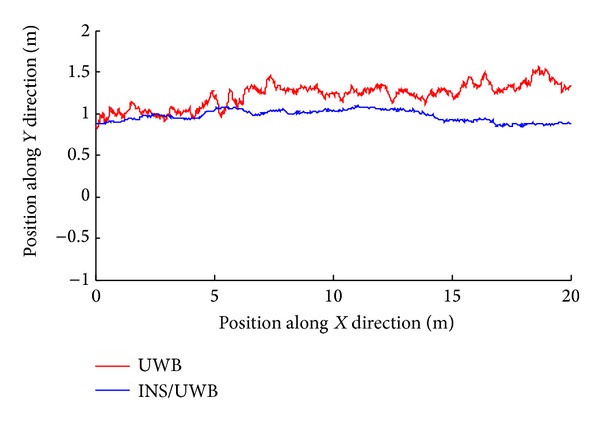
Dynamic tracking performance of position.

**Figure 27:**
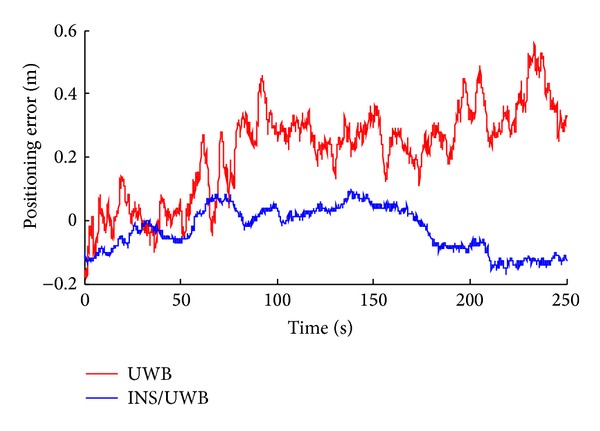
Dynamic tracking error of position.

As the signal frequency of INS is100Hz, and UWB is 10 Hz, system fusion frequency is selected for 10 Hz, so filtering cycle is 0.1 s, and test time is set to 250 s. As shown in Figure 20,* Y* coordinate is set to 1, the initial position *P*_0_ = (0, 1), and the termination position *P*_1_ = (20, 1). The results are shown in Figures 26 and 27.

### 4.3. Result Analysis

We conduct the analysis of experimental results.

**Figure 28:**
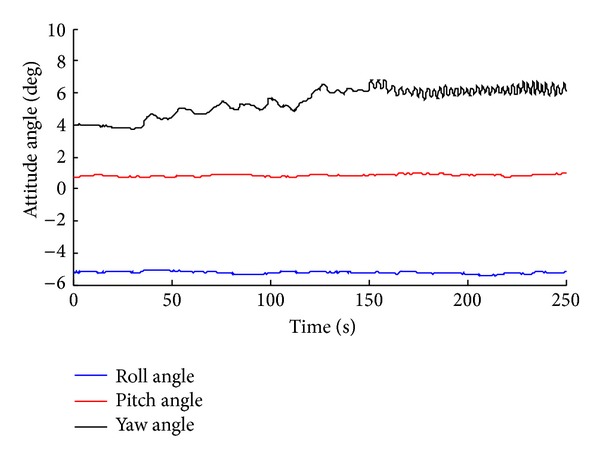
Dynamic tracking performance of attitude angle.

**Figure 29:**
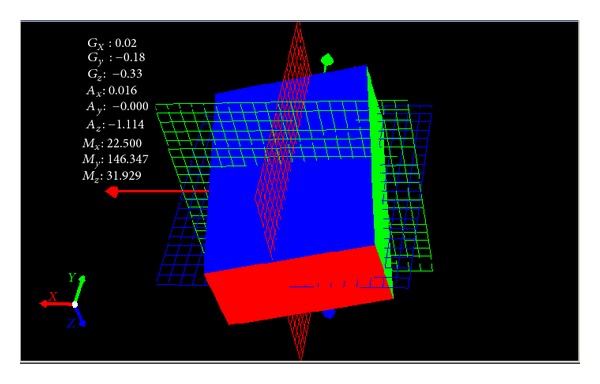
PC interface in the experimental process.

- In the process of static positioning test, as shown in Figures 22 and 23, tracking error range of *x*-axis on the condition of UWB is 0.38 m~1.24 m, the average residual rate is 10.7%, and the confidence level is 89.3%. Then, under the same conditions, tracking error range of INS/UWB is 0.06 m~0.64 m, the average residual rate is 4.6%, and the confidence level is 95.4%. As shown in Figures 24 and 25, tracking error range of  *y*-axis on the condition of UWB is 0.12 m~0.58 m, the average residual rate is 13.2%, and the confidence level is 86.8%. Then, tracking error range of *y*-axis on the condition of INS/UWB is 0.06 m~0.13 m, the average residual rate is 5.1%, and the confidence level is 94.9%.
- In the process of dynamic positioning test, we have designed a set of computer software and PC interface in the experimental process is shown in Figure 29. Dynamic tracking results of attitude angle and position are shown in Figures 26, 27, and 28. Positioning error is Δ*P*_UWB_ = (−0.18 m~0.56 m), Δ*P*_INS/UWB_ = (−0.17 m~0.1 m), the average residual rate is *R*_UWB_ = 7.3%, *R*_INS/UWB_ = 4.8%, and the confidence coefficients are *C*_UWB_ = 92.7% and *C*_INS/UWB_ = 95.2%, respectively.

Results show that integrated navigation system is stable and divergence problem does not exist. At the same time, integrated navigation precision of INS/UWB is better than any independent subsystem.

## 5. Conclusions

- Using INS for positioning independently in a closed environment, the performance of attitude angle tracking is good, but the position error is divergent. This paper proposed a combined method of navigation based on INS/UWB to solve this problem. By analyzing error equation of INS and UWB, respectively, coupled model of INS/UWB was established to realize the fusion of two subsystems.
- On the basis of the coupled model, optimal comprehensive and filtering strategy based on FAKF were proposed. Simulation results show that FAKF has smaller tracking error, which is suitable for INS/UWB integrated navigation data fusion.
- Integrated positioning experiment platform was built according to coal mines closed environment; static and dynamic positioning results show that integrated navigation system based on INS/UWB could track the position and attitude angle of the mobile carrier in real time; and positioning accuracy satisfies the requirement of working condition.

## Figures and Tables

**Table 1:** Drift simulation conditions of inertial drift of the inertial navigation system.

Types of errors	Case 1	Case 2	Case 3
*x*-axis accelerometer drift (ug)	0	10	50
*y*-axis accelerometer drift (ug)	0	10	50
*z*-axis accelerometer drift (ug)	0	10	50
*x*-axis gyroscope drift (deg/h)	0	0.01	0.05
*y*-axis gyroscope drift (deg/h)	0	0.01	0.05
*z*-axis gyroscope drift (deg/h)	0	0.01	0.05
White noise coefficient of deviation	0	0.01	0.05

**Table 2:** Performance comparison between FAKF and KF.

	KF	FAKF
Velocity error range in *x* (m/s)	−0.08~0.05	−0.03~0.02
Velocity error range in *y* (m/s)	−0.12~0.12	−0.07~0.07
Velocity variance in *x*	3.3 × 10^−3^	8.3 × 10^−4^
Velocity variance in*y*	3.1 × 10^−3^	6.1 × 10^−4^
Position error range in *x* (m)	−0.85~0.5	−0.1~0.3
Position error range in *y* (m)	−0.06~0.19	−0.07~0.05
Position variance in *x*	0.0974	0.0048
Position variance in *y*	3.5 × 10^−3^	3.8 × 10^−4^

**Table 3:** Static positioning test data.

*x*_*R*_ (m)	*y*_*R*_ (m)	*x*_UWB_ (m)	*y*_UWB_ (m)	*x*_INS/UWB_ (m)	*y*_INS/UWB_ (m)
1	1	2.24	1.58	1.52	1.13
2	1	2.92	1.31	2.43	1.08
3	1	3.8	1.18	3.31	1.03
4	1	4.74	0.89	4.15	0.94
5	1	5.67	0.91	5.06	0.97
6	1	6.59	1.17	6.08	1.04
7	1	7.43	1.14	6.94	1.08
8	1	8.38	1.14	7.94	1.05
9	1	9.62	0.95	9.1	0.96
10	1	10.77	0.93	10.06	0.97
11	1	11.42	0.9	10.96	0.94
12	1	12.39	1.13	11.98	1.04
13	1	13.67	1.1	13.24	1.03
14	1	14.61	1.09	14.29	1.05
15	1	15.73	1.15	15.29	1.02
16	1	16.81	1.08	16.35	0.97
17	1	17.93	0.94	17.56	0.99
18	1	18.97	1.23	18.58	1.06
19	1	20.05	1.17	19.63	1.08
